# The Role of HO-1 and Its Crosstalk with Oxidative Stress in Cancer Cell Survival

**DOI:** 10.3390/cells10092401

**Published:** 2021-09-13

**Authors:** Shih-Kai Chiang, Shuen-Ei Chen, Ling-Chu Chang

**Affiliations:** 1 Department of Animal Science, National Chung Hsing University, Taichung 40227, Taiwan; g105037017@mail.nchu.edu.tw; 2 The iEGG and Animal Biotechnology Center, National Chung Hsing University, Taichung 40227, Taiwan; 3 Innovation and Development Center of Sustainable Agriculture (IDCSA), National Chung Hsing University, Taichung 40227, Taiwan; 4 Research Center for Sustainable Energy and Nanotechnology, National Chung Hsing University, Taichung 40227, Taiwan; 5 Chinese Medicinal Research and Development Center, China Medical University Hospital, Taichung 40447, Taiwan; 6 Center for Molecular Medicine, China Medical University Hospital, Taichung 404, Taiwan; 7 Department of Biological Science and Technology, China Medical University, Taichung 40402, Taiwan

**Keywords:** heme oxygenase-1, reactive oxygen species, cancers, subcellular localization, mitochondria, nuclei

## Abstract

Heme oxygenases (HOs) act on heme degradation to produce carbon monoxide (CO), free iron, ferritin, and biliverdin. Upregulation of cellular HO-1 levels is signature of oxidative stress for its downstream effects particularly under pro-oxidative status. Subcellular traffics of HO-1 to different organelles constitute a network of interactions compromising a variety of effectors such as pro-oxidants, ROS, mitochondrial enzymes, and nucleic transcription factors. Some of the compartmentalized HO-1 have been demonstrated as functioning in the progression of cancer. Emerging data show the multiple roles of HO-1 in tumorigenesis from pathogenesis to the progression to malignancy, metastasis, and even resistance to therapy. However, the role of HO-1 in tumorigenesis has not been systematically addressed. This review describes the crosstalk between HO-1 and oxidative stress, and following redox regulation in the tumorigenesis. HO-1-regulated signaling pathways are also summarized. This review aims to integrate basic information and current progress of HO-1 in cancer research in order to enhance the understandings and facilitate following studies.

## 1. Heme Oxygenases (HOs) and Oxidative Stress

**Figure 1:**
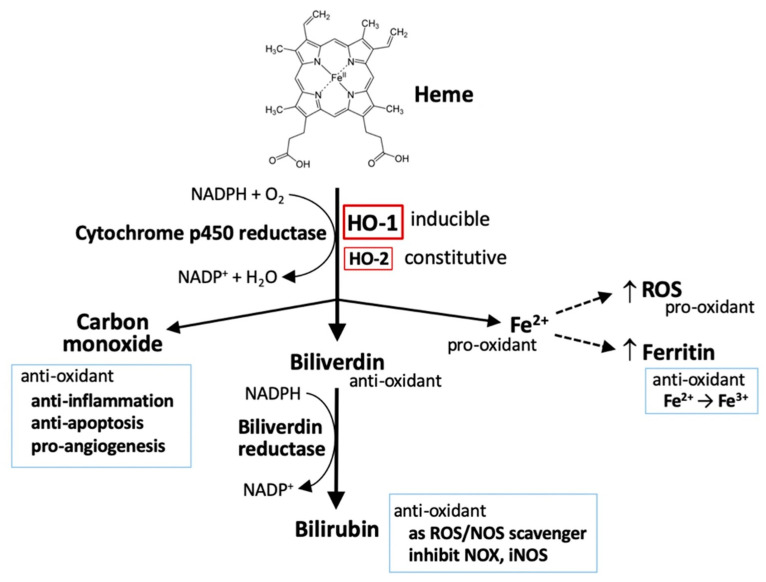
HO-1 and heme metabolism. Heme is degraded by heme oxygenases (HOs), generating biliverdin, carbon monoxide, and ferrous iron (Fe^2+^). Biliverdin is subsequently converted to bilirubin by biliverdin reductase. Both biliverdin and bilirubin act as anti-oxidants by scavenging or neutralizing reactive oxygen species (ROS). Carbon monoxide, a gaseous product, functions in signaling transduction, including the vasodilation of blood vessels, production of anti-inflammatory cytokines, upregulation of anti-apoptotic effectors, and promotion of angiogenesis. Ferrous iron (Fe^2+^) possesses pro-oxidant activity. However, activation of heme oxygenase-1 (HO-1) can upregulate ferritin expression, which binds to ferrous iron and detoxifies its pro-oxidant effect. Ferrous iron increases ROS generation via the Fenton reaction.

HOs are the key-limiting enzymes in heme degradation leading to carbon monoxide (CO), ferrous iron, and biliverdin products. Biliverdin is then rapidly converted to bilirubin by biliverdin reductase (Figure 1) [1,2]. HOs are expressed in a variety of cell types, rendering their broad contribution to cell functions. Currently, three mammalian HO isoforms are identified, namely HO-1, HO-2, and HO-3. HO-1 and HO-2 are identified in human and rats. HO-3 is only found in rats. HO-1 is inducible, synthetically enhanced by pro-oxidant stimuli. HO-1 is encoded by *HMOX1* with a molecular weight of around 32 kDa (also called heat shock protein 32; HSP32) [3]. *HMOX2* is constitutively expressed to encode a 36 kDa HO-2 protein, mainly functioning to maintain the basal heme metabolism and may also play a role in inflammatory responses [4]. *HMOX3* is distinguished as a pseudogene and has no transcript form [5]. 

Oxidative stress is referred as a pro-oxidative circumstance, occurring at the imbalance between oxidants and antioxidants in favor of the oxidants, which has been implicated in governing normal physiological activities and pathological processes. Oxidative stress arises while the generation of reactive oxygen species (ROS) overload the neutralizing capacity of intrinsic antioxidants and antioxidative defenses. Intracellular ROS are mainly generated from mitochondrial electron transport chain, NADPH oxidases (NOXs), and xanthine oxidase [6], as well as, by exogenous stimuli such as electrophiles and ultraviolet light. They are also involved in the regulating of various cellular activities including growth, differentiation, inflammation, infection, ischemia, aging, and disease pathogenesis and progression [7]. Cellular enzymatic and non-enzymatic antioxidants function as an intrinsic defense to prevent oxidant attack and ameliorate oxidative stress. Enzymatic antioxidants include superoxide dismutases, catalase, peroxiredoxins, and glutathione peroxidase (GPX). The most abundant intracellular non-enzymatic antioxidant is glutathione (GSH) [6,7]. 

Despite the fact that ROS are produced as a byproduct in mitochondria biogenesis and intracellular metabolic activities, they are applied in the transduction of cellular signaling or triggering of intracellular defense. Moderately increased level of ROS promotes the systemic defense by inducing adaptive responses to support cell survival, whereas sustained oxidative stress is associated with many pathological conditions such as cancer, metabolic disorders, and neurodegenerative diseases [6,7]. An overload level of ROS can oxidize DNA, RNA, proteins, and lipids, causing irreversible damages and serious oxidative stress that eventually provoke cell death [6,7]. 

In addition to heme metabolism, HO-1 is induced by a broad range of incitements including oxidants, cytokines, growth factors and hormones, heavy metals, and physical cues (ischemia/reperfusion injury and hypoxia/hyperoxia), especially being highly sensitive to pro-oxidant stimuli, such as ultraviolet, heavy metals, inflammatory cytokines, and iron-containing molecules, that contribute to a regulatory network of cell functions [8]. Thus, HO-1 is regarded as a pro-oxidant indicator. Several in vivo studies with HO-1-deficiency have demonstrated a cytoprotective effect of HO-1 in human diseases including systemic inflammation, hemolysis, nephritis, asplenia, nephropathy, and vascular endothelial injury [9,10]. Results from clinical studies further confirmed that HO-1 protects cells by diminishing oxidative stress and inflammation, and maintaining mitochondrial integrity, thereby promoting cell survival [11]. Due to the manner in regulation of iron metabolism [12], HO-1 also play a role in mediating ferroptosis, a newly identified iron-dependent cell death [13,14,15,16]. Since heme degradation generates distinctive metabolites including pro-oxidant ferrous iron and anti-oxidant biliverdin [1,2], HO-1 apparently possesses a dual role either to protect or deteriorate cancer-cell death [8,17,18]. The mechanisms of HO-1 in redox homeostasis and how HO-1 interplays with oxidative stress to regulate tumor progression are addressed in the later sections. 

## 2. The Metabolism of Heme 

### 2.1. Heme

**Figure 2:**
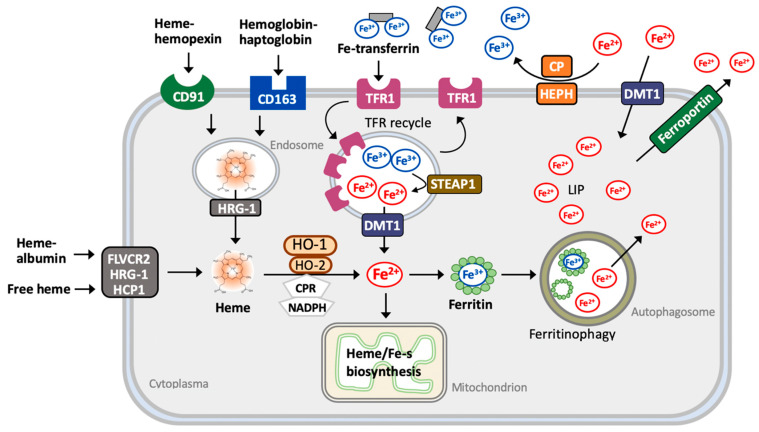
Metabolism of heme. Heme–hemopexin and hemoglobin–haptoglobin complexes are recognized by CD91 and CD163 receptors, receptively, and internalized through endocytosis. Extracellular heme–albumin or free heme is internalized via heme transporters, FLVCR2 (feline leukemia virus C receptor 2), HRG-1 (heme responsive gene-1), and HCP1 (heme carrier protein 1). Internalized heme is then released to transit from the endosome to the cytoplasm via HRG-1 and is further catabolized by HO-1 and HO-2, generating Fe^2+^. The uptake of extracellular Fe^3+^–transferrin is mediated by TFR1 (transferrin receptor 1) through the endocytic process into endosomes, during which they undergo acidification to release Fe^3+^. Free Fe^3+^ is further reduced by metalloreductase enzymes such STEAP3 and reduced Fe^2+^ is transported into the cytoplasma via DMT1 (divalent metal transporter 1). Endosomes are recruited to and fuse with the plasma membranes to release both unbound transferrin and TFRs. Cytosolic Fe^2+^ is either utilized directly as a cofactor of enzymatic proteins or transported to the mitochondria for the synthesis of heme, for Fe–sulfur proteins, or is stored by binding with ferritin. Under the catalysis by ferroxidase, ferritin converts Fe^2+^ to Fe^3+^. Ferritin then is transported to the autophagosome to release Fe^3+^, during which Fe^3+^ is reduced to Fe^2+^ and transferred into the cytoplasmic labile iron pool (LIP). Fe^2+^ can be mobilized as described above or further pumped out the cell via the iron exporter ferroportin. Extracellular free Fe^2+^ may enter cells directly through DMT1. Extracellular Fe^2+^ can be oxidized into less reactive Fe^3+^ by ferroxidase ceruloplasmin (CP) and hephaestin (HEPH).

Circulating irons (Fe) are imported into cells in two different forms, namely free iron and heme-containing iron. More than 80% of bioavailable Fe in mammals is contained within heme, a coordinated complex of Fe with porphyrin called ferriprotoporphyrin. Heme is utilized as a gradient for hemeproteins such as hemoglobin (in red blood cells), myoglobin (in muscle cells), and cytochromes. Extracellular free Fe exists in the plasma mainly by binding to transferrin. Non-heme forms of Fe can transit into the heme along the de novo heme synthesis that transfers Fe to the protoporphyrin IX ring [19]. The entry of free heme or non-binding heme, for example, from hemoglobin release is achieved through several mechanisms. Free heme is captured in plasma by hemopexin or albumin to form heme–hemopexin or heme–albumin complexes. Circulating hemoglobin–haptoglobin complexes, as well as heme–hemopexin complexes, are recognized by the transmembrane protein CD163 and receptor CD91, respectively, for the internalization into cells. Internalization of heme–albumin and free heme is mediated by heme transporters including feline leukemia virus C receptor 2 (FLVCR2), heme responsive gene-1 (HRG-1), and heme carrier protein 1 (HCP1). Intercellular heme transferred from endosomes to the cytoplasma is mediated by HRG-1 [19,20] (Figure 2). The Fe–transferrin complex can interact with transferrin receptor 1, transferrin receptor 2, or cubilin for the internalization through endocytosis. Most of the endosomal ferrous iron (Fe^2+^) is reduced into ferric iron (Fe^3+^) by metalloreductase and transported into the cytoplasm by divalent metal transporter1 (DMT1), followed by the reduction of duodenal cytochrome *b* (DCYTB). Intracellular iron either binds to ferritin for storage or is oxidized to ferric iron by hephasestin and thereafter exported to the circulating transferrin by ferroportin [19,20,21] (Figure 2). 

### 2.2. Iron

Cytoplasmic heme is catabolized by HOs to release free iron. Intracellular iron either binds to ferritin for storage or is oxidized to ferric iron (Fe^3+^) by hephaestin, which is exported to circulating transferrin by ferroportin [19,20,21]. Production of ferrous iron (Fe^2+^) can accelerate the Fenton reaction to facilitate the generation of ROS and following both stress and damages. Ferritin is the major iron storage protein, which processes ferroxidase activity to oxidize Fe^2+^ to Fe^3+^ [22]. Heme thus exerts a pro-oxidative and cytotoxic effect due to its iron function group to provoke ROS production and lipid peroxidation [23]. 

Intracellular iron is primarily utilized in the mitochondria for heme synthesis and iron–sulfur clustering, and also in the cytosol and other organelles. Lysosomal iron is recycled from the mitochondria and cytosolic ferritin through the selective autophagic process and through both mitophagy and ferritinophagy, respectively [24]. Increased ferritin degradation elevates cellular Fe^2+^ levels and provokes both ROS production and lipid peroxidation, leading to ferroptosis [24].

### 2.3. Carbon Monoxide (CO)

The HO system contributes to approximately 85% of CO production and accounts for the main source of endogenous CO. CO is a small gas molecule acting as a gasotransmitter in signaling pathways. For example, CO induces soluble guanylyl cyclase to generate cyclin GMP [25], which controls several critical physiological processes such as vasodilation, redox control, and intracellular signaling [26]. CO can render endothelial cells resistant to endoplasmic reticulum (ER) stress by downregulating CCAAT/enhancement-binding protein homologous protein (CHOP) expression and upregulating the Nrf2/HO-1 pathway [27]. CO also mediates the anti-apoptotic effect of HO-1 via p38 MAPK, PI3K–Akt activation, and K^+^ channel inhibition [28,29,30]. Both in vivo and in vitro studies showed that a low dose of CO selectively inhibited the expression of lipopolysaccharide-induced pro-inflammatory cytokine production including tumor necrosis factor (TNF-α), interleukin (IL)-1β, and macrophage inflammatory protein 1β (MIP1β), whereas it increased anti-inflammatory cytokine IL-10 [31]. Additionally, CO regulates the physiological activities through the implication with cytochrome oxidase, cytochrome p450 reductase, inducible nitric oxide synthase, NADPH oxidases, and mitochondrial cytochromes [32,33,34]. CO also exerts a cytoprotective effect by regulating mitochondrial biogenesis and maintaining mitochondrial integrity for normal membrane potential, permeabilization, and the inhibition of mitochondrial pro-apoptotic pathways [35]. 

### 2.4. Bilirubin and Biliverdin

Bilirubin possesses a potent antioxidant and a quick oxidation by H_2_O_2_ back to biliverdin, forming a catalytic antioxidant cycle driven by NADPH and biliverdin reductase [36]. Bilirubin and biliverdin can directly scavenge ROS including singlet oxygen, O_2_^•^^−^, ONOO^−^, and RO_2_ radicals, inhibiting the activity of NADPH oxidase and inducible nitric oxide synthase, and thereby exerting a cytoprotective function [37,38,39]. 

## 3. The Regulation of HO-1 Expression and Activity

### 3.1. Transcriptional Regulation of HO-1

HO-1 expression is regulated at different levels. In most cases, HO-1 is primarily regulated at the transcriptional level. Several transcription factors are found to bind to the promoter regions of *HMOX1* and participate in its transcriptional activity. Most of the transcription factors are stress-responsive, including nuclear factor erythroid 2-related factor2 (Nrf2), BTB domain and CNC homolog 1 (BACH1), the heat-shock factor (HSF), activator protein-1 (AP1), nuclear factor-κB (NF-κB), hypoxia-inducing factor (HIF-1α), and activating transcription factor 4 (ATF4) [40,41]. Several signaling pathways upstream of the activation of transcription factors have been identified to regulate *HMOX1* expression including MAPK, PI3K/Akt, and protein kinases (PKA, PKC, and PKG) [42]. Surprisingly, HO-1 per se can act as a transcription regulator. Under oxidative stress, transfection of HO-1 cDNA or intracellular delivery of pure protein activated its own transcriptional activity, leading to increased transcript and protein levels [43]. 

Nrf2 is the most important regulator of HO-1. Nrf2 transcriptionally regulates a variety of genes involved in the antioxidant defense called phase II genes, including those encoding HO-1, glutathione *s*-transferase, NAD(P)H quinone oxidoreductase 1, γ-glutamylcysteine synthetase, GPX, menadione reductase, peroxiredoxin, sulfiredoxin, UDP-glucuronosyltransferase, etc. Nrf2 also induces gene expressions involved in mitochondrial biogenesis and quality control, such as superoxide dismutase 2 [8,44]. Nrf2 activity is negatively regulated by the association with kelch-like ECH-associated protein 1 (KEAP1) proteins in the cytoplasm via ubiquitination for proteasomal degradation. Upon oxidation, KEAP1 undergoes conformational change and releases Nrf2. Free Nrf2 is more stable and can translocate into the nucleus in which it forms a heterodimer with the small protein Maf to bind to the ARE of target genes such as *HMOX1* [8,45]. BACH1 is a basic leucine zipper transcription factor, forming a heterodimer with Maf family proteins to compete with the ARE site and thus prevent the access of Nrf2 for *HMOX1* expression [46,47]. Intracellular heme can regulate *HMOX1* expression by directly interacting with BACH1 to loosen its interaction with the promoter and therefore promotes Nrf2 access to bind to ARE elements for HO-1 expression [48]. 

The transcript level of HO-1 is also regulated by microRNA (miR) molecules. MiR-217 and miR-377 work together to reduce the mRNA abundance of HO-1 and thereby result in a decrease of HO-1 proteins [49]. Both miR-24 and miR-1225 also upregulate HO-1 by activating the Nrf2 pathway [50,51]. HO-1 mRNA is also post-transcriptionally downregulated by miR-378 [52]. In the kidney injury model, miR-155 and miR-181a were shown to participate in the cadmium-induced immunotoxicity by downregulating HO-1 expression [53]. MiR-494 upregulated HO-1 expression in neuroblastoma cells under oxidative stress [54]. *HMOX1* expression is also associated with gene polymorphism in the promoter regions in which shorter (GT) repeats in the *HMOX1* promoter exhibit a higher transcriptional activity [55]. 

### 3.2. Translational and Post-Translational Regulation of HO-1

Recently, a novel HO-1 splice variant was identified [56]. The un-translation at the exon 3 of the *HMOX1* gene generates a 14 kDa HO-1 protein, which plays a role in modulating telomere length and tumor growth. Some post-translational modifications of HO-1 have been identified, including ubiquitination for degradation, acetylation, phosphorylation [57,58,59], and truncation [49,60]. Several E3 ligases have been linked to HO-1 expression. Mice with the genetic knockout of seven in absentia homolog 2 (SIAH2), a ubiquitin E3 ligase, exhibited elevated HO-1 protein levels due to enhanced protein stability. SIAH2 deficiency also elevated HO-1 mRNA levels by upregulating Nrf2 expression [60]. Ubiquitination by an ER-resident E3 ligase, TRC8, also regulates cellular HO-1 levels [61]. A rapid turnover rate of HO-1 is attributed to the PEST domain [62]. HO-1 lacking the C-terminal transmembrane segment is susceptible to acetylation by p300 and CREB-binding protein histone acetyltransferase (CREBBP) in the nucleus, which is essential for the nuclear translocation of HO-1 to enhance tumor growth and invasiveness [57]. 

## 4. The Crosstalk between HO-1 and Redox Signaling 

### 4.1. HO-1 in the Endoplasmic Reticulum 

**Figure 3:**
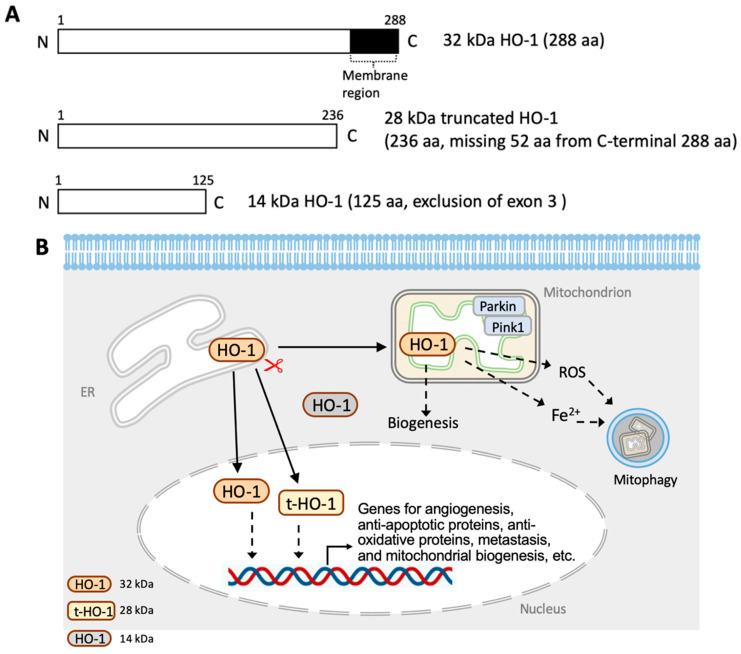
Intracellular traffics of HO-1. (**A**) HO-1 variants. (**B**) The localization of HO-1. After synthesis, the HO-1 protein is delivered to and anchored at the smooth endoplasmic reticulum (ER) membrane. HO-1 can be trafficked to the mitochondria and nucleus under stress or pathological conditions. In the mitochondria, HO-1 affects the mitochondrial biogenesis and dynamics. Mitochondrial HO-1 also increase Fe^2+^ levels and ROS generation, leading to mitophagy. The full-length and truncated form of HO-1 (t-HO-1) are found in the nucleus, in which it regulates gene expressions involved in angiogenesis, anti-apoptotic proteins, anti-oxidative enzymes, metastasis, and mitochondrial biogenesis. The 14 kDa HO-1 variant is retained in the cytosol.

Normally after synthesis, the HO-1 protein is delivered to and anchored at the SER (smooth endoplasmic reticulum) membrane [63]. HO-1 can be trafficked to the mitochondria, nucleus, and rigid domains in the plasma membranes, namely caveolae, under stress or disease conditions [59]. Intriguingly, HO-1 activity is modulated following translocation, after which enzymatic activity is preserved in the mitochondria, vacuole, and plasma membrane, whereas it loses its activity when localized in the nucleus [59,64]. The subcellular localization is associated with post-transcriptional and post-translational modifications. For example, under hypoxia, the completed 32 kDa form of HO-1 in the ER undergoes a cleavage resulting in the nuclear translocation of the 28 kDa truncated form [65]. Ultraviolet light or H_2_O_2_ exposure induces HO-1 expression in a 14 kDa splicing form and is retained in the cytoplasma [56]. HO-1 anchored in the ER through a single transmembrane segment of the C terminus interacts with NADPH, cytochrome p450 reductase, and biliverdin reductase to catalyze heme degradation. The compartmentalization of HO-1 determines its distinctive roles in response to oxidative insults [59,64] (Figure 3).

### 4.2. HO-1 in Mitochondria

Translocation of HO-1 into the mitochondria manifests very severe cellular stress conditions such as oxidative stress, hypoxia, lipopolysaccharide, and smoke [66,67,68]. Additionally, the deletion of the N-terminal of ER-targeting motif increases HO-1 accumulation in the mitochondria [68]. 

Mitochondria are the major generator of ROS, contributing to nearly 90% in cellular ROS production. Within the mitochondrion, electron transport chains act as the major contributor of ROS [6]. Two totally opposite regulatory loops of HO-1 in mitochondrial function have been demonstrated as mitigating or exacerbating oxidative stress. Mice with cardiac-specific overexpression of HO-1 exhibited amelioration of mitochondrial disorganization and dilation of the cardiac sarcoplasmic reticulum induced by doxorubicin. The cardioprotective effects of HO-1 can be attributed to enhanced mitochondrial biogenesis through the upregulation of nuclear respiratory factor 1 (NRF1), peroxisome proliferator-γ coactivator 1 activated receptor 1 α (PGC1α), and mitochondrial transcription factor A (TFAM) [69]. Additionally, HO-1 also acts on the mitochondrial quality control. Under hyperoxia, the heart from cardiomyocyte-specific *HMOX1* knockout mice showed a suppression of the PGC-1α–NRF1 axis and morphologically were characterized by swelling and low density of the mitochondria that were linked to the disturbance in LC3-II-regulated autophagy and Pink1/Parkin2-mediated mitophagy [70]. 

Mitochondrial localization of both intact HO-1 and *N*-terminal truncated HO-1 induced higher ROS production and caused a loss of heme aa-3 and cytochrome *c* oxidase activity in COS-7 cells [68]. Meanwhile, an increase of mitochondrial recruitment of autophagy markers LC3 and Drp-1 was also observed, suggesting an increased mitophagy or autophagy. Additionally, oxidative stress induction and mitochondrial dysfunction by mitochondrial-targeted HO-1 have been identified in anti-cancer agents. For example, an isoflavone anti-cancer agent, ME-344, can bind to HO-1 and promote HO-1 translocation from the SER to the mitochondria, where it alters mitochondrial protein profiles, leading to an interference in tumor cell redox homeostasis and mitochondrial function [71]. BAY117085, a NF-κB inhibitor, was shown to promote ROS generation and mitochondrial localization of HO-1, leading to mitophagy and ferroptosis in breast cancer cells [15]. 

### 4.3. HO-1 in the Nucleus

Nuclear translocation of HO-1 is particularly sensitive to cellular stress [64,65]. Two nuclear forms of HO-1 are identified, namely the 32 kDa intact form and 28 kDa *C*-terminal truncated HO-1 (t-HO-1). Hypoxia or hemin stimuli tends to elicit the nuclear translocation of t-HO-1 deriving from the proteolytic cleavage in SER [65]. Since *C*-terminal-truncation diminishes the oligomerization of HO-1, t-HO-1 shows a very sluggish activity as compared to the intact HO-1 [72]. The *C*-terminal deletion mutant of HO-2 also promotes its nuclear localization [73]. Nuclear HO-1 is able to modulate the activity of transcription factors independent of its enzymatic activity in heme degradation. Both nuclear HO-1 and t-HO-1 increase the transcriptional activity of AP-1, AP-2, and Brn-3, whereas they abolish NF-κB activity [65]. It is noteworthy that the nuclear translocation of HO-1 is highly associated with the malignant progression in many types of cancer such as head and neck squamous cell carcinomas and lung cancers [74,75]. Overexpression of t-HO-1 was shown to promote cell proliferation, migration, and invasion of HeLa and H1299 cells, and thus enhance tumorigenesis [74]. Intriguingly, despite quietly distinctive roles of t-HO-1, both full-length HO-1 and t-HO-1 have been shown to prevent cell death under oxidative stress by H_2_O_2_ induction [65], whereas increased HO-1 expression by hemin treatment exerted an anti-apoptotic effect, but t-HO-1 enhanced cell death [69]. In clinical case studies, the nuclear translocation of HO-1 is associated with cancer progression and poor prognosis in prostate cancer and oral carcinoma [75,76] which contributes to the chemotherapeutic resistance in chronic myelogenous leukemia [77] and myeloma cells [78]. 

## 5. The Contradictory Role of HO-1 in Tumorigenesis

Tumorigenesis is a complicated process, characterized by several stages including mutation, cell transformation hyperproliferation, genome instability, immortalization, angiogenesis, epithelial–mesenchymal transition, and metastasis. ROS is proposed to act as a major regulator in tumorigenesis [79]. As an oxidative stress response, not surprisingly, HO-1 is expressed in a broad range of cancer types such as lymphosarcoma, adenocarcinoma, hepatoma, glioblastoma, melanoma, prostate cancer, and pancreatic cancer [64,80]. The correlational relationship between ROS/HO-1 and tumorigenesis has been discussed in several reviews [64,80,81]. 

### 5.1. HO-1 Deficiency or Mutation in Tumorigenesis

In normal cells, HO-1 is critical in maintaining cellular redox homeostasis by scavenging ROS to prevent DNA damage. Naive *HMOX1*^–/–^ mice exhibit an excessively dysfunctional γ-H2AX foci [82]. The stimuli of genotoxic stressors or irradiation in HO-1-deficient cells caused a loss of ataxia-telangiectasia-mutated (ATM)/ataxia telangiectasia Rad3-related (ATR) proteins and breast cancer 1 proteins (BRCA1), leading to a significant increase of dysfunctional γ-H2AX foci and DNA damage. HO-1 induction or exposure to CO induced the homologous recombination-mediated DNA repair through ATM/ATR in *HMOX1*^–/–^ mice, suggesting the role of HO-1 in DNA-repair signaling [82]. Moreover, in *Mdr2*^−/−^ mice for chronic liver inflammation and inflammation-induced tumor development, administration of HO-1-inducer, CoPP, increased CD8^+^ T cell numbers, reduced DNA damage in liver macrophages of aged mice, and moreover delayed and suppressed tumor growth [83]. 

Pharmacological inhibition and genetic knockdown of HO-1 was shown to potentiate hemin-triggered ROS generation and oxidative DNA damage, and the results were more profound in human colonocyte epithelial cells than those observed in the colorectal cancer cell line [84]. The cytoprotective role of HO-1 also acts at the mitochondrion as observed in skin cells under radiation exposure [85]. HO-1 with the G143H mutant was shown to enhance diethylnitrosamine-induced liver injury and accelerate the tumorigenesis and progression of tumor growth, accompanied with an enhancement of ROS production, hepatocyte damages, and inflammatory IL-6 production [86]. Under hypoxia, induction of HO-2 expression in endothelial cells increased the association with polysomes to enhance the translation of transcripts, allowing cells to maintain a steady level of HO-2 against apoptosis [87]. 

### 5.2. HO-1-Regulated Proliferation and Development of Cancer Cells 

In human primary head and neck squamous cell carcinoma (HNSCC) specimens, HO-1 was found with a high level of expression, mostly localized in the nuclei in cancerous tissues than non-tumor tissues. In a mouse model of squamous cell carcinoma and HNSCC, cytoplasmic HO-1 expression was observed in pre-neoplastic lesions, whereas nuclear HO-1 expression was identified in tumor tissues, suggesting the role of nuclear HO-1 in promoting tumor growth [75]. Moreover, nuclear localization of HO-1 is associated with malignant performance in colorectal, prostate, and breast cancer [88,89,90]. However, in some human astrocytoma and oligodendroglioma subtypes, tumor malignancy is paralleled with total cellular HO-1 levels not compartmentalized HO-1 in the nuclei [91]. In fact, HO-1 are involved in substantial mechanisms to support the proliferation and invasiveness of the tumor. HO-1 can act as a BCR/ABL-dependent survival factor in chronic myeloid leukemia [92]. It also participates in the hepatocyte growth factor-induced c-Met–Ras signaling-enhanced proliferation of renal cell carcinoma [93]. In human colon cancer cells, namely HT-29, HO-1 mediates EGFR–Src–NF-κB signaling to promote cell proliferation [94].

In tissue-associated leukocytes, HO-1 is highly expressed in monocytic cells in the microenvironments surrounding the tumor, rendering the cells differentiated into tumor-associated macrophages (TAMs) [95]. Iron metabolism plays a pivotal role in the microenvironments for tumor cell growth, especially by TAMs [96]. TAMs are the main population of immune cells in tumor microenvironments, in which they acquire diverse phenotypes and functional profiles to differentiate into pro-inflammatory (M1) or anti-inflammatory (M2) states. M2-like TAMs are found in the hypoxic and necrotic areas of tumor microenvironments, which are characterized by high levels of ferroportin and low levels of ferritin, presenting an enhanced phenotype of iron-release. Accordingly, M2-like TAMs are capable of supporting tumor cell proliferation, angiogenesis, and metastasis via promoting vascularization in the tumor microenvironments [96]. In the prostate cancer xenograft mouse model, deletion of HO-1 in macrophages suppressed tumor growth, in which HO-1-derived CO from TAMs’ downregulated E-cadherin expression to mediate tumor pathogenesis and progression [97]. 

### 5.3. HO-1-Regulated Angiogenesis of Cancer Cells 

Angiogenesis is necessary for continued growth, invasion, and metastasis of solid tumors [98]. HO-1 overexpression in pancreatic cancer cells markedly promoted tumor angiogenesis and accelerated the occurrence of metastasis in a lung colonization model [99]. Angiogenesis by HO-1 is likely mediated by the upregulation or activation of proangiogenic factors such as VEGF and stroma cell-derived factor-1 (SDF-1) [100,101]. Nuclear translocalization of HO-1 increased VEGF expression and secretion in prostate cancer cells [102]. Treatment of ZnPP, a HO-1 inhibitor, suppressed HIF-1α expression and VEGF production, accompanied by the enhanced proliferation of HCT-15 cells, suggesting that the angiogenesis for tumor growth is mediated by HIF-1α and VEGF [103]. VEGF-enhanced angiogenesis by HO-1 was further shown as operating at the upregulation of cyclin A1, cyclin E1, and cyclin-dependent kinase 2 activity, as well as vimentin to enhance the proliferation of human endothelial cells [104]. In addition, induction of HO-1 expression attenuated high glucose-mediated ER stress and downstream events in endothelial cells, including oxidative stress, activation of inflammatory responses, and apoptosis, as well as enhanced VEGF-A expression [105]. In addition, CO, the metabolite by HO-1, can promote VEGF expression by increasing HIF-1α content at the translational level and post-translational stabilization of the HIF-1α protein [106]. 

The lung metastasis resulting from subcutaneous tumors or circulating tumor cells was significantly repressed in mice bearing bone marrow HO-1^+/–^ as compared to those in wild type mice [107], suggesting that HO-1 expression in hematopoietic cells impacts tumor colonization at the metastatic site. The mechanism was further attributed to chemoattractant-induced myeloid cell migration through p38 kinase signaling and to tumor cell transendothelial migration through the vascular endothelial growth factor, IL-10, and STAT3 activation [107]. In a similar manner, mice intravenously injected with HO-1-overexpressed melanoma cells, namely B16-HO1, were characterized by augmented vascularization and a higher level of vascular endothelial growth factors in the tumor, whereas a lower level of serum TNF-α but a higher level of soluble receptor TNF-RI were observed. HO-1 overexpression apparently accelerated B16 melanoma cell metastasis in the lungs and resulted in a low survival rate [108]. 

Despite the numerous reports regarding the pro-tumor effects of HO-1, overexpression of HO-1 in non-small cell lung carcinoma upregulates the tumor-suppressive factors, miR-378 and p53 expression; downregulates angiopoietin-1 and mucin-5AC (MUC5AC); suppresses cell proliferation and migration; and rather unexpectedly diminishes angiogenic potential CO to act as a mediator of HO-1 effects [52]. Conversely, miR-378 overexpression downregulated HO-1 and p53 expression but increased VEGF and MUC5AC expression, cell proliferation, and migration [52]. 

### 5.4. HO-1-Regulated Metastasis of Cancer Cells

The epithelial to mesenchymal transition plays an important role in cancer progression from initiation, primary tumor growth, invasion, dissemination, and metastasis to colonization as well as resistance to therapy [109]. The analysis of the microarray dataset from clinical biopsies showed that *HMOX1* expression levels significantly increase in glioma grade IV brain biopsies when compared to grade I, II, and III. Additionally, the expression level of HO-1 in glioma grade IV brain biopsies was correlated to the chemotaxis gene expression [110]. In A2780 and SKOV-3 ovarian cancer cells, ROS scavengers, namely *N*-acetyl-_L_-cysteine and HO-1 inhibitor ZnPP, were shown to relieve ROS production and autophagy, and ameliorate cell migration and invasion by reversing the epithelial–mesenchymal transition [111]. Genetic silence of GRP78, an ER stress response protein, enhanced the metastasis by promoting vimentin and decreasing E-cadherin expression through the Nrf2/HO-1 pathway in HT-29 colon cancer cells [112]. Additionally, the deficiency of GRIM-19, an essential subunit of the mitochondrial MRC complex I, accelerated gastric cancer metastasis through the ROS–Nrf2–HO-1 axis [113]. The role of HO-1 in cancer progression involves cell cycle regulation. Mice treated with HO-1 inhibitor ZnPP had a reduced thyroid cancer xenograft growth and diminished cyclin D1 and Ki-67 expression [114]. The results were further confirmed in vitro, showing that ZnPP induced a G_0_/G_1_ arrest of cell cycle, accompanied by decreased cyclin D1 and CDK4, and an increase of p21 and p27 expression [114]. Moreover, ATF4 and Nrf2 can work together to transcriptionally activate HO-1 to ameliorate oxidative stress and prevent both anoikis and lung metastasis [41]. These results reveal that both HO-1 and ROS crosstalk with each other in coordinating subcellular compartmentalization, related effectors, and cascadings to contribute the epithelial–mesenchymal transition. 

The anti-tumor effects by mitigating metastasis were also observed in NCI-H292 lung mucoepidermoid carcinoma cells. Nrf2 overexpression-derived HO-1 inhibited NCI-H292 cell proliferation and migration, and downregulated oncogenic miR-378, multiple matrix metalloproteinases (MMP-1 and MMP-9), and inflammatory IL-1β expression [115]. Furthermore, the Notch1/Slug pathway was found to mediate the antitumor role of HO-1 in mouse mammary carcinoma [116]. 

**Figure 4:**
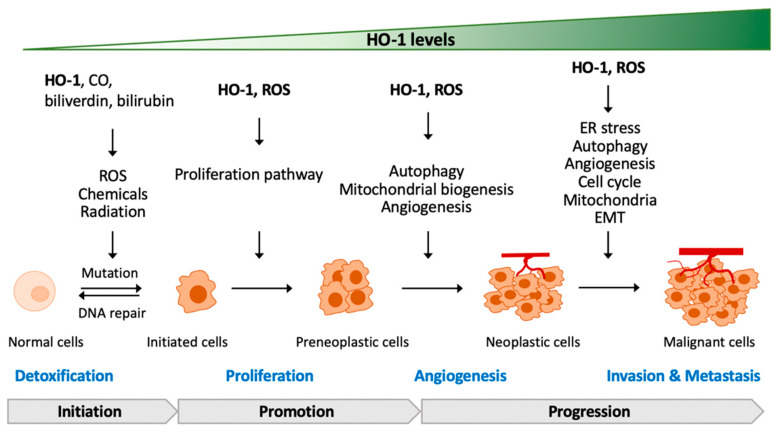
HO-1, ROS, and tumorigenesis. HO-1 can suppress the transformation of cancer cells via preventing ROS-induced mutation. Once the tumorigenesis process is triggered, ROS and HO-1 may serve as the mediator roles to support the proliferation, angiogenesis, invasion, and metastasis of cancer cells. Abbreviation: EMT, epithelial–mesenchymal transition.

In summary, in normal cells, ROS or chemicals/radiation-increased ROS can cause DNA damage and mutation, which may further lead to the cell transformation to cancer cells. In response to increased ROS, HO-1 is thereby raised to neutralize ROS and eliminate DNA damage that reduce the chances of acquired mutation. Once tumorigenesis is initiated, more fuels are required to support cancer cell proliferation. In the proliferation from initiated cells, the increase of both ROS and HO-1 regulates the mitochondrial biogenesis, which may co-work with autophagy and redistribute to the metabolic system, allowing for the adaption of enhanced fuel requirements for the fast growth of cancer cells. For high-fuel demand following the fast growth, angiogenesis is necessary to establish the transportation network. In this stage, ROS and HO-1 communicate with the nuclei to upregulate pro-angiogenesis factors such as VEGF and promote the proliferation of vascular epithelial cells. For metastatic colonization, ROS and HO-1 co-work to regulate the protein expression responsible for the epithelial–mesenchymal transition and cell cycle. Based on clinical observations, ROS and HO-1 levels increase gradually along the malignancy. It is reasonable to propose that ROS and HO-1 assist each other to contribute to tumorigenesis via serving as the communicators in linking with the ER, mitochondria, and nuclei to set up an optimal environment for cancer cells (Figure 4). 

## 6. HO-1-Drived Resistance against Therapy

Provocation of intracellular ROS is often observed in chemotherapy and radiotherapy. The excessive ROS-mediated killing considerably contributes to anti-cancer effects. However, sustained oxidative stress tends to drive cancer cells to acquire resistance against chemotherapy or radiation therapy. ROS-mediated mechanisms for the acquired resistance were found operative at the ER stress induction, autophagy disturbance, cell cycle arrest, and reprogramming of the epithelial–mesenchymal transition [117,118]. Several chemotherapy studies suggested that following ROS rising, increased cellular HO-1 levels serve as an initiator in the acquirement of resistance, including etoposide, doxorubicin gemcitabine, cisplatin, lapatinib, sapatinib, and radiotherapy [119,120,121,122,123,124].

The HO-1-related autophagic process involved in chemoresistance development was observed in studies with sapatinib, lapatinib, doxorubicin, pharmorubicin, or radiation treatment [119,120,121,122,124]. HO-1 disturbs autophagy by regulating Beclin-1, p62, and LC3B-I/II expression, which in turn contributes to the chemotherapeutic resistance [121,123,124] and radio-resistance [125]. In addition, the anti-oxidant effects of HO-1 allow for cancer cells to overcome ROS-induced DNA damage and apoptosis in response to chemotherapy and radiotherapy [125,126]. The HO-1-regulated therapeutic resistance is summarized in Table 1.

**Table 1:** The effects of HO-1-contributed resistance to chemotherapeutic agents and radiation therapy.

Therapeutic Treatment	Cancer Types	HO-1 Status	Resolution or HO-1 Regulator	Reference
BIX-01204 (G9a inhibitor)	KG leukemia stem cells	Induced PERK–autophagy–ROS–HO-1	PERK inhibition (shRNA, GSK2606414) or autophagy inhibition (Bafilomycin A1) to inhibit Nrf2/HO-1 and increase ROS	[127]
Bortezomib (BTZ)	Neuroblastoma: HTLA-231 and MDA	Increased Nrf2 and HO-1	ZnPP co-treatment enhanced sensitivity of BTZ-mediated apoptosis	[128]
Bortezomib (BTZ)	Neuroblastoma: HTLA-230	Activated Nrf2 to increase HO-1 gCLM, xCT, and GSH	HO-1 siRNS sensitized BTZ-induced cell death, all-trans retinoic acid (Nrf2 inhibitor) reversed BTZ-increased HO-1, gCLM, xCT, and GSH, and sensitized Bortezomib-induced cytotoxicity	[129]
Bortezomib (BTZ)	Multiple myeloma (U266, KMS26 SKM-M1, and MM1S)	Induced ER stress, ROS generation, and upregulated nuclear HO-1	E64d prevented nuclear localization of HO-1 and increased BTZ sensitivity	[78]
Bortezomib (BTZ)	Parent U226 and bortezomib-resistant U266	Theioredoxin reductase regulated Nrf2 and HO-1	ZnPP restored BTZ-mediated apoptosis	[130]
Cisplatin	Ovarian cancer: SKOV-3 and CAOV-3 Human ovarian cancer tissues	Induced Sirtuin 5–Nrf2–HO1 pathway to inhibit ROS generation Higher Sirtuin-5 expression	Sirtuin 5 siRNA sensitized cisplatin-induced ROS and DNA damage	[126]
Cisplatin	Hepatoma cells: HepG2, 97H, and SMMC7721 HepG2 xenograft	Increased HO-1 expression	ZnPP co-treatment increased ROS, caspase-3 activity, and apoptosis ZnPP enhanced cisplatin-inhibited tumor growth	[131]
Cisplatin and pisrarubicin	Hepatoblastoma: HepG2 Human hepatoblastoma specimens (cisplatin and pirarubicin)	Induced EGFR–AKT/ERK–HO-1	EGFR inhibitor (AG1478) and siHO-1 sensitized cisplatin and pirarubicin-induced cell death	[132]
Cytarabine	Leukemia HL-60 and cytarabine-resistant HL-60 (HL-60R) Chemotherapy relapsed samples	HL-60R cells have higher HO-1 expression compared to parental HL-60 Higher HO-1 and HIF-α expression	HO-1 siRNA sensitized cytarabine-induced apoptosis in HL-60R cells	[133]
Doxorubicin (DOX)	Breast cancer: MDA-MB-231, and -MB-231	Induced Src–STAT3–HO-1 Increased HO-1 induced a cytoprotective autophagic flux and increased both Beclin-1 and LC3-I/II	SiRNA of Src and STAT3 sensitized DOX-induced cell death and DOX-increased HO-1, and prevented HO-1-upregulated Beclin-1 and LC3-I/II	[121]
Doxorubicin Vinblastine Radiation	Lung adenocarcinoma cells: A549	HRP-3–Nrf2–HO-1–ROS–p53–PUMA pathway mediated chemoresistance and radioresistance	HRP3 siRNA enhanced sensitivity of doxorubicin, vinblastine, and radiation-induced apoptosis	[134]
5-Fluoracil (5-FU)	MDR1-overexpressed colon carcinoma (HCT-116/R)	HCT-116/R cells expressed higher expression of HIF-1F, Nrf2, and HO-1, as well as increased NOX2 activity and ROS compared to parental cells	NOX inhibitor (HDC) and Nrf2 inhibitor (ML-385) enhanced 5-FU-induced apoptosis	[135]
5-Fluorouracil (5-FU)	Pancreatic cancer, CPFAC and BxPC-3	Increased HO-1 (higher NQO1 and SOD2) Higher EMT marker (Nanog, Oct4, CD133, and ABCG2)	Nrf2 siRNA increased sensitivity of 5-FU-mediated cytotoxicity	[136]
5-Fluorouracil (5-FU)	Colorectal cancer: SNUC5 and 5-FU-resistant SUNC5 (SNUC5-5-FUR)	ISNUC5-5-FUR exhibited increased ROS–Nrf2–HO-1 compared to parental cells	shRNA of Nrf2 or HO-1 enhanced sensitivity of 5-FU-mediated apoptosis of SNUC5-5-FUR cells and tumor inhibition in SNUC5-5-FUR xenograft mouse	[137]
Gemcitabine Radiation	Pancreatic cancer cells: Panc-1, Mla PaCa-2, SU8686, and Colo 357	Increased HO-1 expression	HO-1 siRNA enhanced sensitivity to Gemcitabine and radiation-mediated cell death	[119]
Gemcitabine Radiation	Urothelial carcinoma: T24 and MGHU3	Increased HO-1 expression	ZnPP co-treatment enhanced sensitivity of gemcitabine or radiation-mediated apoptosis	[120]
NMS E793	A375 melanoma cells	Upregulated ER stress response protein IRE1α, ERO-1, GRP78, and CHOP Upregulated HO-1	SnMP (HO-1 inhibitor) co-treatment induced higher ER stress, increased ROS, and promoted apoptosis	[138]
Pharmorubicin	MDA-MB-231, MCF-7 breast cancer cells	Induced PI3K-AKT-HO-1-autophpagy (LC3-I/II)	HO-1 siRNA sensitized pharmorubicin-mediated reduced chemoresistance	[124]
Radiation	Lung adenocarcinoma cells: A549	Increased HO-1 and ROS levels	HRP-3 knockdown Inhibited Nrf2/HO-1 Enhanced ROS	[134]
Low-dose radiation	Lung adenocarcinoma cells: A549	Induced ROS–autophagy–Nrf2-HO-1	NAC (ROS scavenger) blocked autophagy and Nrf2/HO-1; Nrf2 knockdown or ZnPP treatment reversed resistance to radiation	[125]

The co-treatment of chemotherapy/radiotherapy with HO-1-regulating agents may serve as a way to attenuate therapy resistance. SnMP (tin mesoporphyrin), a HO-1 inhibitor, significantly enhanced ER stress and apoptotic effects by the HSP90 inhibitor NMS E973 in A375 melanoma cells [138]. The histone methyltransferase G9a has been identified as a potential target for the epigenetic therapy of acute myeloid leukemia. Both PERK and Nrf2 inhibitors synergistically enhanced the effects of the G9a inhibitor BIX-01294 on cell apoptosis, consistent with the suppressed HO-1 expression, increased p38 MAPK activation, and ROS generation in acute myeloid leukemia cells [127]. Inhibition of HO-1 nuclear translocation by E64d disturbed the genomic stability and sharpened the sensitivity to cytotoxic bortezomib in multiple myeloma [78]. Furthermore, HO-1 mediated the sensitization of miR-200c to sorafenib and imatinib in clear-cell renal cell carcinoma cells [139]. Similarly, exposure to CO sensitized prostate cancer cells to doxorubicin through growth arrest and apoptosis induction [76]. The mechanisms were shown to operate at mitochondrial functionality due to CO targeting including oxygen consumption and ROS generation, leading to mitochondrial collapse and consequently mitotic catastrophe [76]. Pentacarbonyl iron (Fe(CO)5) as a CO producer was shown to expedite mitochondrial metabolic exhaustion for ATP synthesis, inhibit ATP-dependent drug efflux, reverse the resistance of doxorubicin, and induce apoptosis in MCF-7/ADR tumors [140]. 

## 7. HO-1 Commands the Lifespan of Cancer Cells

### 7.1. HO-1 and Apoptosis

Apoptosis is a type 1 programmed cell death in response to the extrinsic death receptor signaling and intrinsic mitochondrial pathways. The extrinsic death pathway is initiated from the death ligands binding to the death receptors, leading to the assembly of the death-inducing signaling complex (DISC) and activation of effector caspases, namely caspase-8, -9, and -3. ROS can enhance the DISC complex by downregulating the cellular FLICE-inhibitory protein (c-FLIP), a completer of DISC. The intrinsic mitochondrial pathway is activated by the alteration of mitochondrial permeability and cytochrome *c* leakage, which ultimately results in the formation of apoptosome and activation of caspase-9 [141]. ROS also induces apoptosis by promoting cysteine oxidation and downregulating anti-apoptotic protein Bcl-2, which increases mitochondrial permeability and thereby enhances apoptosis [142,143]. In addition, ROS can trigger cell apoptosis by inhibiting the expression of negative regulators of autophagy (TORC1) and enhancing the formation of LC3-dependent autophagosomes [143]. Overexpression of HO-1 protected renal cancer cells from rapamycin and sorafenib-induced apoptosis through upregulation Bcl-xL expression and both Beclin-1 and LC3B-II downregulation [144]. 

In human esophageal squamous cell carcinoma tumors, the level of ROS is negatively correlated to HO-1 expression and apoptosis. Genetic knockdown of HO-l increased ROS production, activated caspase-9 and -3, and induced apoptosis in TE-13 cells and Eca109 cells [145]. c-Met activation was shown to protect renal cancer cells from sorafenib-induced ROS and following cytotoxicity [146]. The underlying mechanisms were shown through the enhanced Nrf2–HO-1 signaling to eliminate ROS production, promote anti-apoptotic protein expression (Bcl-2 and Bcl-xL), and inhibit caspase-3 activity [146]. Similarly, a diminishing of ROS and JNK–c-Jun signaling was shown to mediate the protective effects of HO-1 in β-amyloid-induced apoptosis in neuroblastoma cells [147]. Conversely, apatinib, a new oral tyrosine kinase inhibitor that targets VEGF signaling, can inhibit the Nrf2–HO-1 pathway to exhaust the cellular glutathione reservoir and thereby significantly elevate intracellular ROS levels, leading to apoptosis and autophagy in ovarian cancer cells [148]. These studies suggested the protective role of HO-1 in cell apoptosis involving the provocation of ROS. 

### 7.2. HO-1 and Ferroptosis

Ferroptosis is a newly identified programmed cell death characterized with ROS overload, iron accumulation, and lipid peroxidation-dependence, which can be moderated by ROS scavengers, iron chelators, and lipid peroxidation inhibitors [13]. Ferroptosis tends to alter cell morphologies, including the shrinkage of mitochondria with increased membrane density and vanishing of mitochondrial cristae [13](Dixon). Inhibition of system Xc^–^ and GPX4 expression or activity primes the ferroptotic process [13,149]. System Xc^–^, an amino acid anti-transporter located on the phospholipid bilayers of the membranes is composed of two subunits, namely SLC7A11 and SLC3A2, and mediate the uptake of cysteine. Intracellular cysteine is taken up into cells and further reduced to become cysteine for glutathione synthesis. GSH is then used as the substrate to eliminate ROS and reactive nitrogen species (RNS) carried by GPXs. GPX4 converts GSH to oxidized glutathione (GSSG) and further reduces the cytotoxic lipid peroxides to corresponding alcohols. Inhibition of GPX4 expression can cause lipid peroxidation and ferroptosis [149]. Voltage-dependent anion channels (VDAC) are transmembrane channels located on mitochondrial outer membranes, which are involved in the regulation of ions and small molecules across the outer membrane and thus play an important role in ferroptosis [13,150]. Iron can trigger lipid peroxidation, resulting in the disruption of membrane integrity [151]. Erastin induces ferroptosis via targeting on system Xc^–^ and VDAC [13]. The HO-1 inhibitor ZnPP has been shown to prevent Erastin-induced ferroptosis in HT-1080 fibrosarcoma cells, whereas the HO-1 inducer hemin and CO inducer CORM both promoted Erastin-induced ferroptosis but not by biliverdin and bilirubin [14]. Erastin-induced HO-1 expression was enhanced by hemin and CORM, suggesting that HO-1 is required for ferroptosis induction [14]. Furthermore, small molecules such as BAY117085 and withaferin A were shown to promote the Nrf2 expression required for the upregulation of HO-1 in cancer cell death [15,16]. The activation of Nrf2 by BAY117085 was further shown through increased ROS production and GSH depletion, which in turn promoted HO-1 expression as well as mitochondrial and nuclear translocation, leading to mitochondrial dysfunction and mitophagy [15,16]. 

Although Nrf2-mediated HO-1 expression contributes to ferroptosis [15,16], enhanced Nrf2 expression can potentiate the resistance against ferroptosis. During the induction of ferroptosis by artesunate, Nrf2 is activated and serves as a resistant factor. Nrf2 deficiency by genetic knockdown or by the inhibitor trigonelline reversed the resistance to ferroptosis in artesunate and cisplatin-resistant head and neck cancer cells [152]. Co-treatment with β-elemene and cetuximab increased the sensitiveness to ferroptosis inducers in KRAS mutant colorectal cancer cells [153]. The induction of ferroptosis was characterized by iron-dependent ROS accumulation, GSH depletion, excessive lipid peroxidation, and upregulation of HO-1 and transferrin [153]. The combination of siramesine, a lysosomotropic agent, and lapatinib, a dual tyrosine kinase inhibitor, synergistically induced cell death in breast cancer through ferroptosis in glioma cells U87 and lung adenocarcinoma cells A549. Co-treatment of siramesine and lapatinib enhanced iron release from lysosomes, increased the degradation of HO-1, and downregulated HO-1 expression [123]. An increase of HO-1 by CoPP reversed the ferroptotic effects of the siramesine and lapatinib combination [154]. The progression of ferroptosis is also ameliorated by bilirubin due to the inhibition of lipid peroxidation but not through ROS generation [154]. In summary, HO-1 is proposed to act as a mediator in conducting ferroptosis due to the iron release-promoting effects that may promptly increase ROS production. The pro-oxidant status further enhances HO-1 activity, forming a feedback loop in amplifying the accumulation of ROS that is apparently unable to be eliminated by intracellular antioxidants, and thus causes overwhelmed lipid peroxidation to trigger cell ferroptosis. 

## 8. Conclusions and Perspectives

**Figure 5:**
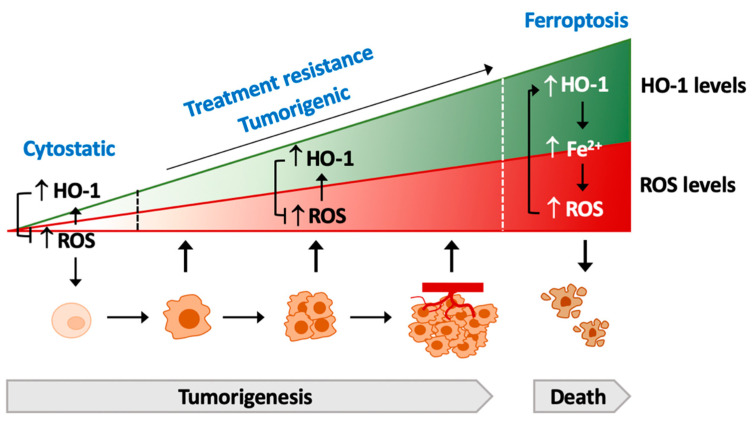
Model of the crosstalk of HO-1 and ROS-mediated tumorigenesis. HO-1 exerts a cytoprotective effect by scavenging ROS to prevent the cell transformation. During tumorigenesis, HO-1 serves as a protector to ameliorate the deteriorated effects of increased-ROS to cancer cells, which supports the proliferation and expansion, as well as the acquiring, of therapeutic resistance. Conversely, excessive activation of HO-1 increases labile Fe^2+^, leading to ROS overload and the death of cancer cells, namely ferroptosis.

HO-1 exerts multiple cytoprotective functions in association with angiogenesis and its counteraction in the detrimental effect of oxidative stress, critical for the survival of tumor cells. The high expression of HO-1 is found in a wild range of cancer types and correlates with the poor prognosis and resistance against chemotherapy, suggesting the inhibition of HO-1 as a therapeutic target for chemotherapy [155]. However, with the pro-oxidant property of HO-1 under pro-oxidative status [14,15,16], HO-1 is implicated to serve a potential role in promoting cancer cell death in cancer therapy. There are several concerns that should be addressed. Due to the variant expression and dual function of HO-1 expression during tumorigenesis, it is a dilemma to balance the HO-1-inhibiting or HO-1-enhancing therapy. Promoted HO-1 expression may help normal cells dealing with oxidative stress that prevents cell transforming. In the initiation step of oncogenesis, HO-1 upregulation may allow cancer cells to overcome the deleterious effect of ROS overload derived from the accelerated metabolic rate for cell proliferation. A higher oxidative stress can sustain the activation of HO-1 in cancer cells and thus favor cancer clonal expansion, invasion, and metastasis. Aggressive ROS generation serves as a part of anti-cancer effects in chemotherapy and radiotherapy. However, the anti-oxidative property of HO-1 may result in the resistance against cancer treatments. Intriguingly, the pro-oxidative effects of HO-1 may cause an irreversible oxidative damage via increased ferrous-amplified oxidative stress, resulting in lipid peroxidation and cell ferroptosis (Figure 5). Therefore, the corresponding stages of tumorigenesis or tumor growth alone with the treatment of HO-1-inhibiting or HO-1-promoting agents become a critical issue. Furthermore, the combination treatment of chemotherapy with HO-1-inhibiting agents may be able to mitigate the resistance against the therapeutic agents.

This review highlights the compartmentalization of HO-1 acting at various levels to regulate cell functions. HO-1 is localized in the nucleus in malignant cells with a low enzymatic activity in the moderately differentiated tumors, which correlates with the relatively worse clinical outcomes. Mitochondrial localization of HO-1 seemingly benefits the mitochondrial biogenesis and quality control, which facilitate cancer cells to maintain redox homeostasis. In contrast, it may cause mitochondrial dysfunction in response to excessive oxidative cues. Prevention of the nuclear translocation of HO-1 may elevate the therapeutic efficacy of HO-1-regulating agents. The controversial role of HO-1 suggests a complexity in the regulatory network in cancer biology. The elucidation of the crosstalk between HO-1 and oxidative stress provides a better strategy to dictate HO-1 as a target for cancer therapy. 

## Abbreviations

BACH1, BTB domain and CNC homolog 1; CHOP, CCAAT/enhances-binding protein homologous protein; CO, carbon monoxide; CORM, CO-releasing molecule; ER, endoplasmic reticulum; GSH, glutathione; GPX, glutathione peroxidase; HO, heme oxygenase; HIF-1α, hypoxia-inducing factor-1α; IL, interleukin; KEAP1, kelch-like ECH-associated protein 1; LC3, microtubule-associated protein 1A/1B light chain 3; miR, microRNA; NOX, NADPH oxidase; Nrf2, nuclear factor erythroid 2-related factor 2; ROS, reactive oxygen species; SER, smooth endoplasmic reticulum; SOD, superoxide dismutase; TAMs, tumor-associated macrophage; t-HO-1, C-terminal truncated HO-1; TNF, tumor necrosis factor; and VEGF, vascular endothelial growth factor.
